# Marginal bone loss around oral implants supporting fixed versus removable prostheses: a systematic review

**DOI:** 10.1186/s40729-020-00217-7

**Published:** 2020-06-03

**Authors:** Babak E. Saravi, Maria Putz, Sebastian Patzelt, Amir Alkalak, Sara Uelkuemen, Martin Boeker

**Affiliations:** 1 Department of Prosthetic Dentistry, Center for Dental Medicine, Medical Center—University of Freiburg, Freiburg, Germany; 2 Department of Prosthetic Dentistry, Center for Dental Medicine, Medical Center—University of Freiburg, Freiburg, Germany; 3 Institute of Medical Biometry and Statistics, Faculty of Medicine, Medical Center—University of Freiburg, Freiburg, Germany

**Keywords:** Oral health, Dentistry, Marginal bone loss, Fixed prosthesis, Removable prosthesis, Overdenture, Marginal bone loss, Implant-supported

## Abstract

**Aim:**

The aim of this systematic review was to evaluate and compare the marginal bone loss (MBL) around implants of fixed (FISP) versus removable implant-supported prosthesis (RISP).

**Material and methods:**

This review was conducted according to the PRISMA guidelines. A systematic search of the literature on Web of Science and Ovid (MEDLINE) was conducted in March 2019 to identify randomized controlled trials/quasi-randomized trials, prospective and retrospective studies written in German and English. Two reviewers screened the identified papers for eligibility and performed an independent data extraction. The Newcastle-Ottawa Scale was used to evaluate the level of evidence of the included studies.

**Results:**

The search resulted in 2577 studies, of which 42 were selected for full-text evaluation. Finally, six studies were included in qualitative analyses, reporting results from 248 participants (81 FISP versus 167 RISP). Five of the included studies were prospective and one study was retrospective. MBL was highest in the first year after implant placement and ranged from 0.17 ± 0.07 mm to 2.1 ± 1.6 mm in FISP and from 0.22 ± 0.55 mm to 2.5 ± 2.7 mm in RISP. After 4 years, there was no statistically significant difference between the groups; MBL ranged from 0.36 ± 0.22 mm to 1.5 mm in FISP and 0.56 ± 0.45 mm to 1.4 mm in RISP. Of the six included studies, two each were rated as good quality, fair quality, and poor quality.

**Conclusion:**

Fixed and removable implant-supported prostheses seem to have similar long-term outcomes regarding marginal bone loss. However, the evidence provided in this systematic review is limited due to the poor quality of two of the included studies. Future studies with study designs specified to the topic of this review are necessary to provide clear information about marginal bone level alterations in modern implant therapy.

## Introduction

Since the investigation of the osseointegration of oral implants by Brånemark in the 1950s and 1960s, implant-supported restorations became a widespread alternative to removable prostheses [1, 2]. Both then and now, edentulism is a serious health problem involving functional, aesthetic, phonetic, and psychological problems [3, 4]. Although the Fifth German Oral Health Study illustrated a decrease in edentulism among 65 to 74-year olds from 24.8% in 1997 to 12.4% in 2015, therapy concepts are still needed which focus on the resulting disabilities [5]. As the epidemiological findings indicate a decrease in edentulism, treatment concepts for partial edentulism and single-tooth replacement re also considered important in order to meet patient requirements.

The success of implant rehabilitation relies on the integration of the implants in hard and soft tissues. Marginal bone loss (MBL) is, therefore, a critical factor affecting the clinical outcome [6, 7]. While moderate MBL of < 0.2 mm per year are generally accepted as within the limits of a normal physiological process, excessive MBL, in particular in the first year after implant insertion, is associated with an increased risk of peri-implantitis and tissue collapse affecting not only the survival rates of oral implants but also aesthetics especially in the anterior visible zone [8]. Multifactorial reasons for early excessive MBL are assumed; however, they are not fully understood. The main theories have been the infection theory, supported mainly by periodontists, and the overload theory supported by prosthodontists/restorative dentists [9]. However, there is clear evidence that combined factors affect MBL and a single-minded explanatory model for bone loss is not acceptable [9, 10]. In a systematic review, Qian et al. found no evidence that overloading alone represents the incriminating factor behind marginal bone resorption around oral implants [9]. Furthermore, aseptic foreign body reactions and strong immune and inflammatory reactions of the individual host immune system play a key role in MBL progression [9, 11, 12]. MBL primarily occurs in the early stage after implant placement. While an early MBL of 1.0–1.5 mm during the first year has long been assumed as normal, newer data shows losses of 0.459–0.55 mm in the first year after implant insertion [7, 13–16]. Surgical trauma, occlusal overload, peri-implantitis, microgap, biologic width, and implant crest module have been reported to be positive causative factors for early implant crestal bone loss [17]. Albrektsson et al. and Smith and Zarb proposed bone loss of less than 0.2 mm annually after the implant’s first year of service as one criterion for implant success [7, 14]. In a recent study, an early MBL of > 0.44 mm after the first 6 months of prosthetic loading was suggested as a risk factor for the progression of peri-implant bone loss [18].

For implant-supported restorations, the fixed screw-retained and cemented as well as removable restorations are established treatment options [19, 20]. For removable implant-supported prostheses, the number of supporting implants, prosthesis design, and the attachment type are important factors for outcome analysis [21, 22]. Survival rates for removable or fixed implant prostheses were found to be similar when patients were matched for bone quantity and quality, implant number, implant length, and splinting [19, 23].

To the authors’ knowledge, the long-term effect on the marginal bone level around implants of implant-supported fixed and removable restorations has not been compared and systematically evaluated in a review. Thus, the aim of this systematic review of the available literature was to evaluate the effect of the restoration type (fixed or removable) on the marginal bone level of oral implants.

## Material and methods

This review was conducted based on the guidelines of the transparent reporting of systematic reviews and meta-analysis (PRISMA) and in accordance with the Cochrane handbook for systematic reviews of interventions [24, 25].

### Search strategy

The research question in the PICO format was formulated as follows [26]:

P (participant): partially and fully edentulous patients

I (intervention): FISP

C (comparison): RISP

O (outcome): marginal bone loss

Based on the PICO summary, blocks were built and completed with variants and synonyms to create a specific search strategy [27].

Two independent reviewers performed a systematic search in Web of Science (Clarivate Analytics (US) LLC., Philadelphia, PA, USA; http://.webofknowledge.com) and MEDLINE by using the Ovid Web Gateway Internet Interface (Ovid Technologies, Inc, New York, NY; http://gateway.ovid.com). The search was conducted in March 2019 and was restricted to studies published in English or German.

Free-text terms were used to search the Web of Science database (Table 1). Medline via OVID was searched with a combined medical subject heading and a free-text term search strategy (Table 2). Then, text-word-truncation was applied to retrieve all forms of the search terms and Boolean logical operators were used to combine the search results.

**Table 1:** Electronic free-text term search in Web of Science

#1 TS=(((impl* OR overdentur* OR maxillofacial OR “maxillo-facial”) NEAR/5 (prosthes* OR fixed OR permanent OR removable OR restorat* OR reconstruct*)))	
#2 TS=(((bone* OR osteo*) NEAR/5 (alveolar OR maxill* OR mandib* OR jaw OR bucc* OR “peri-implant*“ OR periimplant* OR marginal) NEAR/5 (loss OR resorption OR defect* OR density OR atroph* OR distraction OR lyses OR osteolys* OR osteonecro* OR necrosis OR volume)))	
#3 TS=((predict* OR score* OR observe*) OR (trial OR random* OR therapeutic*))	
#4 #2 AND #1	
#5 #4 AND #3

**Table 2:** Combined Medical Subject Headings (MESH terms) and free-text term search in MEDLINE via OVID

1 exp Dental Prosthesis/ 2 exp Dental Implants/ 3 exp Dental Prosthesis Retention/ 4 exp Jaw, Edentulous/rh, su [Rehabilitation, Surgery] 5 Exp Bone-Implant Interface/	**Patient population and intervention**
6 Periodontal Atrophy/ 7 exp Alveolar Bone Loss/ 8 exp Gingival Recession 9 exp Periodontal Attachment Loss/ 10 exp Alveolar Process/rh [Radiography] 11 exp Mandible/su [Surgery] 12 exp Maxilla/su [Surgery|	**Outcome**
13 1 or 2 or 3 or 4 14 5 or 6 or 7 or 8 or 9 or 10 or 11 or 12 15 13 or 14 16 randomized controlled trial.pt. 17 controlled clinical trial.pt. 18 randomized.ab. 19 placebo.ab. 20 clinical trial as topic.sh. 21 randomly.ab. 22 trial.ti.	**Study design**
23 16 or 17 or 18 or 19 or 20 or 21 or 22 24 exp animals/ not humans.sh. 25 23 not 24 26 15 and 25	

### Eligibility criteria

The evaluation of titles and abstracts as well as full-text analysis was conducted according to the eligibility criteria listed in Table 3.

**Table 3:** Eligibility criteria

Inclusion criteria	Exclusion criteria
• Studies including human subjects in good general health (**patient/population**) • Partially or fully edentulous patients (**patient/population**) • No restriction on age or gender (**patient/population**) • Restorations supported by osseointegrated implants with no restriction on material, design, number, location, surgical technique and loading protocol (**intervention**) • Intervention and comparison group, FISP versus RISP (**comparison)** • Marginal bone loss around maxillary and mandibular implants, with no restriction on the location (**outcome**) • Validated, standardized measurement methods to investigate MBL (**outcome**) • Randomized controlled trials (RCTs), quasi randomized controlled trials (qRCTs), prospective and retrospective studies (**study design**) • Publications in English or German with no restriction on date (**study design)**	• Unpublished work • Patients with diabetes, periodontal diseases, pregnancy, osteoporosis, heavy smokers and studies in which >25% of the patient population were smokers • Studies with less than 15 patients • Patients with augmented bone • Tooth-implant-supported restorations • Single-tooth implant restorations • Studies including animals and in vitro studies

### Study selection and data extraction

The selection of studies was a two-step process. In the first step, two reviewers (B.S. and M.P.) independently screened the titles and abstracts to determine which studies met the inclusion criteria. In the second step, they assessed the eligibility of the selected studies based on a full-text analysis. While screening the publications, the following parameters were extracted into an extraction chart: author, complications of the inserted implants and restorations, mean age of groups, mean MBL, observation period, number of patients participating, number of patients in the groups, number of implants, study design, dental status, survival and success rate, year of publication. Search agreement between the two reviewers was evaluated with the Cohen’s Kappa (k) test. Any disagreements were resolved by a discussion with a third reviewer.

### Risk of bias

The risk of bias and quality analyses of the studies were performed by the two reviewers independently. The Newcastle-Ottawa Scale (NOS) was used to analyze the included studies [28].

Studies can have score values between 0 and 1 in the “selection” and “exposure/outcome” categories and between 0 and 2 in “comparability”. As there are four items in the “selection” category, one item in the “comparability” category and three items in the “exposure/outcome” category, the maximum score according to the NOS is nine. A score of “0” was awarded when the criterion was not satisfied, a score of “1” was awarded when the criterion was satisfied, and for “comparability”, a score of “2” was awarded when the criterion was satisfied using a validated method.

The NOS score was used to categorize overall study quality as either good quality (> 7), fair quality (6–7), or poor quality (< 6).

### Statistical analysis

Descriptive analyses were performed to calculate weighted means and standard deviations (SD) using standard spreadsheet software (Excel for Mac 2011, version 14.5.2, Microsoft, Redmond, WA, USA).

## Results

### Screening process and study characteristics

**Fig. 1:**
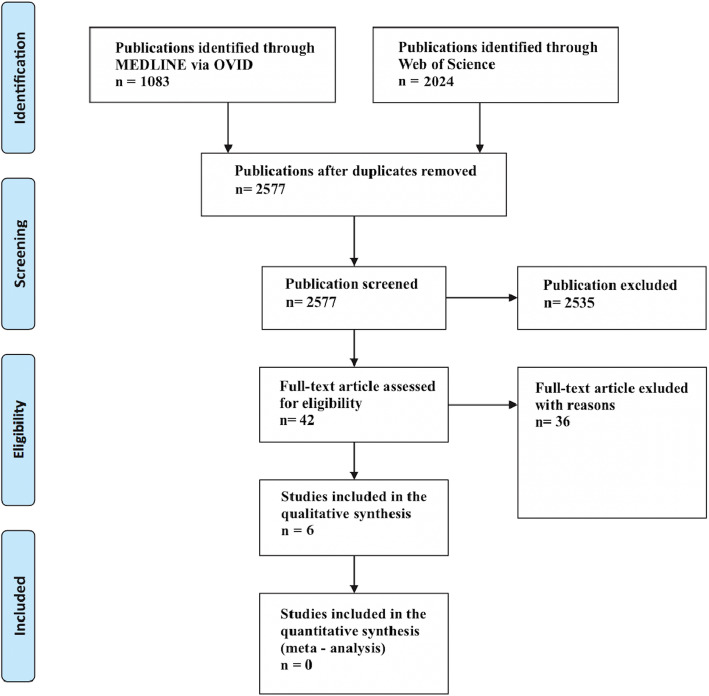
PRISMA flow diagram of the screening and selection process. Reasons for exclusion are shown in Table 4

The search resulted in 1083 studies from Medline and 2024 studies from Web of Science (Fig. 1). A total of 530 were duplicates. After scrutinizing the titles and abstracts, 2535 studies were excluded due to not meeting the inclusion criteria, leaving 42 publications for full-text analyses. After examining the full texts, 36 papers were excluded because they did not meet the defined inclusion criteria (Table 4). The remaining six studies were included in the systematic review. Five of the included studies were prospective and one was retrospective. The number of patients ranged from 30 [29] to 74 [30] with a mean of 43.7 ± 14.7. The number of investigated oral implants ranged from 60 [29] to 235 [30] with a mean of 160.2 ± 56.9, supporting a mean of 20.2 ± 13.8 FISP and 21.2 ± 7 RISP. The number of implants inserted per patient ranged from 5 to 6 for FISP and from 2 to 4 for RISP. One study [31] did not report the overall number of implants and three studies [29–31] did not report the number of implants inserted per patient for the FISP and RISP group (Table 5**)**. The *k* values for potential article inclusion (titles and abstracts) and selected studies were 0.79 and 0.88, respectively, indicating excellent agreement between the reviewers.

**Table 4:** Reasons for exclusion

Reason for exclusion	Number of excluded studies
Review	3
No data on MBL	5
No comparison of MBL between fixed and removable prostheses	16
Inclusion of patients with diabetes, periodontal diseases, augmented bone, pregnancy, osteoporosis, heavy smokers or studies in which > 25% of the patient population were smokers	10
Tooth-implant-supported restorations	1
Single-tooth implant restoration	1

**Table 5:** Characteristics of studies included in this review

Author (year)	Study design/follow-up (years)^a^	Patients	Age (mean±SD/range)	Region	Status	Type of prosthesis	No. of implants Overall FISP/RISP (no. of implants per patient)
Palmqvist et al. 1996 [29]	PS (not randomized)/4,9	*n* = 30	NR^b^	Maxilla	Fully edentulous	15 FISP 15 RISP	Overall: 60 FISP: NR^b^ RISP: NR^b^
Cune et al. 1996 [30]	RS/1	*n* = 74	55.1 ± 12.1 18.5–83.1	Maxilla Mandible	Fully edentulous	10 FISP 50 RISP	Overall: 235 FISP: NR^b^ RISP: NR^b^
Makkonen et al. 1997 [32]	PS (not randomized)/5	*n* = 33	39–75	Mandible	Fully edentulous	13 FISP 20 RISP	Overall: 155 FISP: 5-6 RISP: 4
Tinsley et al. 2001 [33]	PS (randomized)/5	*n* = 48	37–80	Mandible	Fully edentulous	21 FISP 27 RISP	Overall: 181 FISP: 5 RISP: 2-3
Raghoebar et al. 2003 [34]	PS (not randomized)/3	*n* = 40	56 30–70	Mandible	Fully edentulous	10 FISP 30 RISP	Overall: 170 FISP: 5 RISP: 4
Quirynen et al. 2004 [31]	PS (not randomized)/10	*n* = 37	59.3 36–85	Mandible	Fully edentulous	12 FISP 25 RISP	NR^b^

^a^PS: prospective study, RS: retrospective study

^b^NR: not reported

### Assessments of quality and risk of bias

**Fig. 2:**
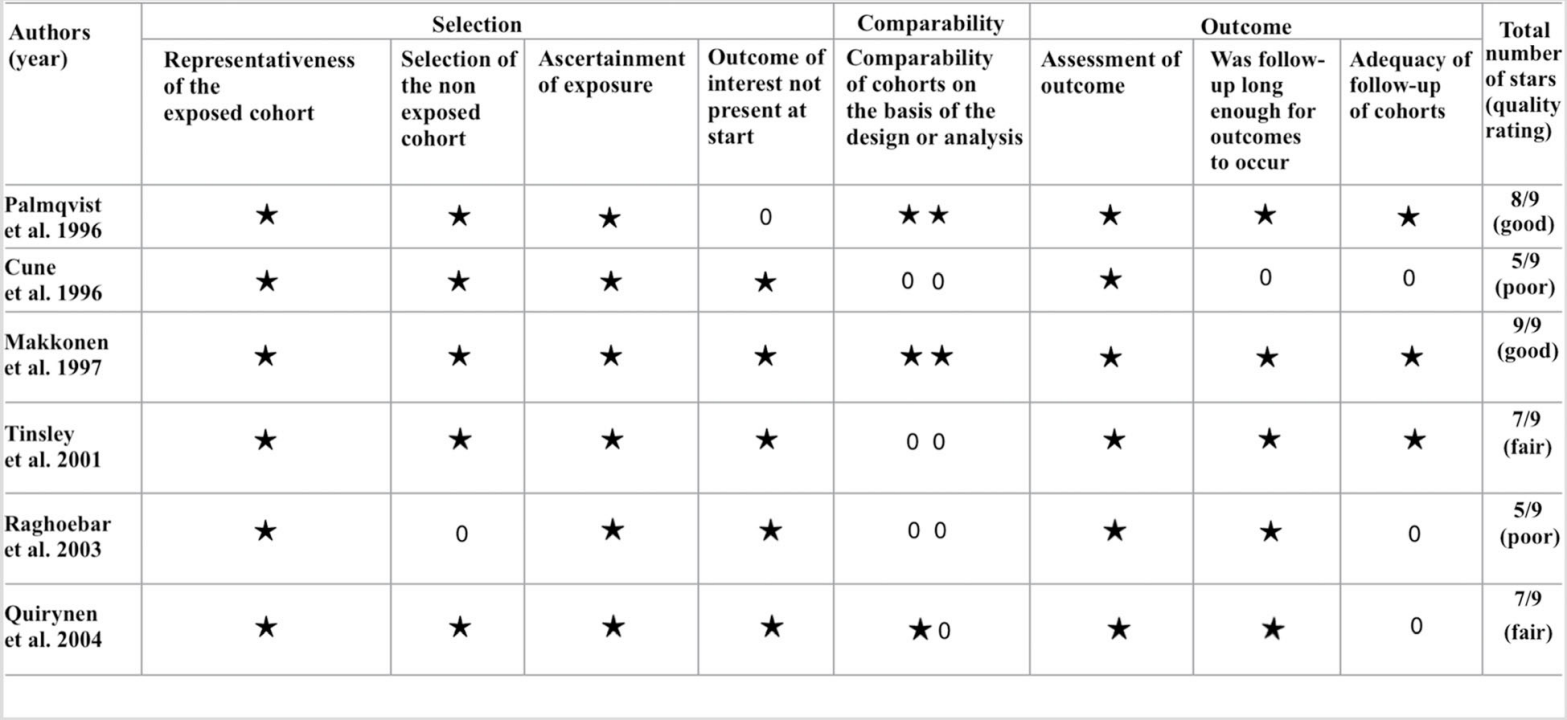
Assessments of quality and risk of bias via the Newcastle-Ottawa Scale. For a better visualization, stars have been used to represent the scores in the categories

Two studies [30, 34] were rated as overall poor quality, mainly because of their potential risk of bias regarding the comparability domain. Two studies [31, 33] were considered fair quality and two [29, 32] as good quality (Fig. 2).

### Data synthesis

The observation period of the selected studies ranged from 1 to 10 years with a mean of 4.8 ± 2.7 years. MBL measurements were evaluated using detailed narrow beam radiography (DNB) [29, 32] or long cone intraoral radiography [30, 31, 33, 34]. In four studies [30–33], the region of implant-abutment connection was used as the baseline reference for the MBL measurements in mm. Measurements were taken mesially and distally to determine bone level changes. In one study [29], the authors used a scoring system using the threads of the implant as a measuring scale for mesial and distal measurements, providing no mm values. However, the data was used for comparability analyses between FISP and RISP regarding MBL in the given observation period. One study [34] did not provide information about the measurement technique.

MBL was most significant in the first year after implant placement, ranging from 0.17 ± 0.07 mm to 2.1 ± 1.6 mm for FISP and 0.22 ± 0.55 mm to 2.5 ± 2.7 mm for RISP (Table 6). Palmqvist et al. used a scoring system, thus conversion to mm was not possible. Weighted arithmetic means of the score were calculated for each of the given years and listed in Table 6.

**Table 6:** MBL outcomes (FISR versus RISR) in the included studies

Authors (year)	Year 1 (FISP/RISP)	Year 2 (FISP/RISP)	Year 3 (FISP/RISP)	Year 4 (FISP/RISP)	Year 5 (FISP/RISP)	Year 6 (FISP/RISP)	…Year 10 (FISP/RISP)	Conclusion
Palmqvist et al. 1996^a^		1.25 ± 0.43 1	1.5 ± 0.5 3.75 ± 1.3	1.63 ± 0.42 3 ± 0.17	1.4 ± 0.66 1.5 ± 0.67	1.58 ± 0.76 1.04 ± 0.2		No significant difference in MBL between FISR and RISR after 4 years
Cune et al. 1996^b^	2.1 ± 1.6 2.5 ± 2.7							NR^c^
Makkonen et al. 1997^b^	0.17 ± 0.07 0.31 ± 0.34	0.19 ± 0.17 0.45 ± 0.4	0.25 ± 0.16 0.53 ± 0.38	0.3 ± 0.18 0.53 ± 0.42	0.36 ± 0.22 0.56 ± 0.45			No statistically significant difference in MBL between FISP and RISP after 4 years. MBL statistically significantly higher (*p* < 0.04) in RISP compared with FISP after 3 years.
Tinsley et al. 2001^b^	1 0.5	1.1 0.6	1.2 0.8	1.4 1	1.5 1.4			Initially more MBL in FISP. After that slightly more rapid MBL in RISP group. At the end of the 5th year MBL in both groups remarkably similar.
Raghoebar et al. 2003^b^	0.36 ± 0.60.22 ± 0.55	0.47 ± 0.62 0.39 ± 0.48						No significant difference in MBL between RISP and FISP after 3 years (*p* > 0.3)
Quirynen et al. 2004^b^							0.73 0.86	MBL between FISP and RISP not significantly different after 10 years.

^**a**^Author used a scoring system, thus conversion to mm was not possible. Weighted arithmetic means were calculated for each of the given years

^b^Values are mean cumulative bone loss (mm) described as a distance from a fixed baseline reference point

^c^NR: not reported; author did not comment on the MBL outcome FISP vs RISP

In all studies, the data suggested a similar outcome between FISP and RISP regarding MBL at the end of the observation period. According to the available data, MBL seems to initially be higher in RISP but does not differ between the groups after 4 years. Two studies [33, 34] observed initially higher MBL in FISP. However, this observation was either not significant for all observation periods [34] or no information was provided regarding statistical significance for any of the given observation periods [33]. Cumulative implant survival rates and the number of implant failures in the included studies are shown in Table 7. Palmqvist et al. and Quirynen et al. did not provide information regarding implant survival rates and the number of implant failures. For the superstructures, Makkonen et al. reported a survival rate of 100% after 5 years and a limited number of complications represented in both groups, FISP and RISP. None of the complications resulted in any lasting problems. In the fixed prosthesis group provided by Tinsley et al., five patients (5/21) required a remake. Reasons were soft tissue proliferation beneath the cantilever (two patients), wear of acrylic teeth (two patients), and fracture of the superstructure (one patient). In the overdenture group, nearly half of the patients (13/27) required a remake. Raghoebar et al. listed the frequency of reported complications without differentiating between FISP and RISP. From the twenty-four patients reporting complications, ten had a clip out of overdenture and two had a clip fracture. Quirynen et al. and Palmqvist et al. did not provide information regarding the longevity of the superstructure in the overdenture and the fixed prosthesis group.

**Table 7:** Implant survival characteristics in the included studies

Author(year)	Implant survival at the end of the study^a^	Implant loss/failing implants	Observation period for surviving implants	Comments
Palmqvist et al. 1996 [29]	NR	NR	4.9 ± 1.2 years range: 2–6 years	No data provided for implant survival and implant loss
Cune et al. 1996 [30]	94.5 ± 2.7%	Overall: 24 FISP: 5 RISP: 19	513.3 ± 182.8 days	Implant loss most frequently in the maxilla (18/24)
Makkonen et al. 1997 [32]	Overall: 98.7% FISP: 100% RISP: 97.4%	Overall: 2 FISP: 0 RISP: 2	5 years	Both lost implants judged to be non-osseointegrated at the time of abutment connection
Tinsley et al. 2001 [33]	FISP: 71% ± 4.37 RISP: 71% ± 4.94	Overall: 13 FISP: 7 RISP: 6	72 months	Thirteen implants had extensive bone loss > 4 mm and were defined as “failing implants” according to the authors criteria
Raghoebar et al. 2003 [34]	Overall: 93% FISP: 94% RISP: 93%	Overall: 12 FISP: 3 RISP: 9	3 years	One implant failed prior to loading. Eight implants failed after four weeks and three implants failed after one year of observation.
Quirynen et al. 2004 [31]	NR	NR	10 years	No data provided for implant survival and implant loss.

^a^Values are shown as cumulative implant survival rates

^b^NR: not reported

Different attachment types were used in the RISP groups among the included studies. The overdenture (RISP) patient population examined by Quirynen et al. [31] was divided into three groups: a “bar” group, provided with an egg-shaped Dolder bar; a “magnet” group with two open-field magnets; and a “ball” group with two ball attachments. Data on MBL was only provided overall, however, not for each separate group. Palmqvist et al. provided no data regarding attachment types, as the overdenture group (RISP) consisted of a subpopulation of consecutive patients from an earlier study [29, 35]. In the overdenture group (RISP) investigated by Cune et al., implants were splinted by a bar, either with or without extension. The overdenture group (RISP) investigated by Raghoebar et al. received four implants with bar attachments and standard abutments. Tinsley et al. did not provide information regarding attachment type in the overdenture group. However, the patients in this group were treated with two or three implants. The overdenture group (RISP) examined by Makkonen et al. received four implants anterior to the mental foramina. The implants were attached with clips to a Dolder bar linked to the implants. Due to the heterogeneity of the available data, a meta-analysis was not performed.

## Discussion

Despite great achievements in global oral health, edentulism remains a major and irreversible problem affecting quality of life. In providing adequate therapy options for these patients, information regarding outcomes plays a key role in decision-making. Thus, the aim of the present review was to systematically evaluate the differences in MBL—an important factor for the long-term success of oral implants—between FISP and RISP [6, 7]. The included studies were published in peer-reviewed scientific journals and judged to be reliable. Nevertheless, an assessment of the potential risk of bias was performed using the Newcastle-Ottawa Scale to indicate the level of evidence for the main research question.

For long-term observations, the available data suggest that both therapy options result in a similar amount of MBL. Reported MBL after 1 year of loading was 2 mm to 3.2 mm for RISP (depending on the attachment type and implant number) and 1.5 mm for screw-retained FISP [23, 36, 37]. The difference in MBL between screw-retained and cemented FISP was evaluated in several studies. While Nissan et al. [38], Lemos et al. [39], and Hameed et al. [40] reported greater MBL in screw-retained FISP, Koller et al. [41] and Sailer et al. [42] found that cemented FISP showed more MBL (> 2 mm) than screw-retained restorations. In contrast to these findings, systematic reviews by Brandao et al. [43] and Sherif et al. [44] did not find evidence for a significant difference in MBL between screw-retained and cemented restorations. After 1 year of observation, MBLs reported in the included studies ranged from 0.17 ± 0.07 mm to 2.1 ± 1.6 mm in FISP and 0.22 ± 0.55 mm to 2.5 ± 2.7 mm in RISP. Other authors reported similar findings regarding MBL values after 1 year of observation ranging from 0.1 mm to 1.5 mm [13, 14, 45]. In all included studies providing data from more than 4 years, MBL for RISP and FISP was similar after 4 years [29, 31–33]. Only one included study [30] investigated MBL in both jaws. The values described in mm in four of the included studies focused on osseointegrated implants in the mandible [31–34]. As described in Table 3, Palmqvist et al. used a score system; thus, conversion to mm was not possible. Cune et al. reported MBLs of 2.6 ± 2.1 mm for the maxilla and 1.9 ± 1.5 mm for the mandible. In contrast to previous findings of Quirynen et al. [37], where most subjects with maxillary overdentures showed continuous bone losses of up to 3 mm or more after 53 months of loading, data provided by Palmqvist et al. do not support the theory of continuous bone loss in patients provided with overdentures [29, 37]. However, Palmqvist et al. reported that the poorest bone scores were found in three overdenture patients [29].

This review included data for up to 10 years; thus, the long-term effect of the two different therapy options could be examined. Furthermore, three of the included studies, reporting data from more than 4 years for 100 patients overall, were rated as “good quality”. This underlines the strength of the evidence for the conclusions of this review.

Nevertheless, there are several limitations to this review that need to be addressed. As described by Palmqvist et al., different results for MBL outcomes are likely due to patient selection criteria, length of the placed implants, design of the prostheses, and different operators with different surgical techniques [29]. The previously mentioned factors limit the comparability of different studies. The prostheses’ attachment type is an important confounding variable and is crucial to take into account in interpreting the results. Due to the different attachment types used across the studies, comparisons are limited. Two of the included studies [32, 34] used one attachment type for all overdenture patients. The other studies either did not provide enough information regarding the attachment type [29, 33] or used various attachment types in their overdenture group [30, 31]. No studies provided data on MBL between different attachment types, and therefore, the effect of attachment type on MBL remains unanswered. Furthermore, the timing of the baseline measurements and a description of the measurement technique are important to consider in interpreting the findings. Nevertheless, these crucial details are often not well described. In the present review, two studies were rated of “poor quality” mainly due to a lack of comparability between the intervention groups. For example, Palmqvist et al. matched the control patients (FISP group) with the intervention patients (RISP group) based on observation period, age, and gender, while Raghoebar et al. did not. The reason can be found in these studies’ primary research questions. While Palmqvist et al.’s focus was on marginal bone level alterations in the intervention groups, MBL was not the primary outcome in the other included studies.

The above-mentioned limitations and the low number of relevant studies emphasize the need for better-designed studies of the differences in MBL between FISP and RISP. The authors encourage future investigators to conduct prospective studies according to the guidelines described by the Cochrane handbook for systematic reviews of interventions, with control patients matched for factors affecting MBL outcome [24].

## Data Availability

All data generated or analyzed during this study are included in this published article.
